# Linking human brain functional connectivity to underlying neurotransmission

**DOI:** 10.64898/2026.04.28.721294

**Published:** 2026-05-05

**Authors:** Leon D. Lotter, Golia Shafiei, Daouia Larabi, Abhay Koushik, Ottavia Dipasquale, Mitul Mehta, Mara Cercignani, Arjun Sethi, Neil Harrison, Štefan Holiga, Daniel Umbricht, Igor Yakushev, Suresh Muthukumaraswamy, Anna Forsyth, Joerg F. Hipp, Bratislav Misic, Svenja Caspers, Julian Koenig, Kaustubh R. Patil, Casey Paquola, Simon B. Eickhoff, Juergen Dukart

**Affiliations:** 1 Institute of Neuroscience and Medicine (INM-7: Brain and Behaviour), Research Centre Jülich; Jülich, Germany.; 2 Institute of Systems Neuroscience, Heinrich Heine University Düsseldorf; Düsseldorf, Germany.; 3 Max Planck School of Cognition; Stephanstrasse 1A, Leipzig, Germany.; 4 Penn Lifespan Informatics and Neuroimaging Center (PennLINC), Perelman School of Medicine, University of Pennsylvania; Philadelphia, PA, 19104, USA.; 5 Department of Psychiatry, Perelman School of Medicine, University of Pennsylvania; Philadelphia, PA 19104, USA.; 6 Lifespans Brain Institute (LiBI), Children’s Hospital of Philadelphia and Penn Medicine; Philadelphia, PA, USA.; 7 Department of Clinical and Developmental Neuropsychology, University of Groningen; Groningen, the Netherlands.; 8 Max Planck Institute for Human Cognitive and Brain Sciences; Stephanstr. 1A, Leipzig, Germany.; 9 Olea Medical; La Ciotat, France.; 10 Institute of Psychiatry, Psychology, and Neuroscience, King’s College London; London, UK.; 11 Cardiff University Brain Research Imaging Centre (CUBRIC), Cardiff University; Cardiff, UK.; 12 Roche Pharma Research and Early Development, Roche Innovation Center Basel, F. Hoffmann-La Roche Ltd; Basel, Switzerland.; 13 Xperimed LLC; Basel, Switzerland.; 14 Department of Nuclear Medicine, School of Medicine, Klinikum Rechts der Isar, Technical University of Munich; Munich, Germany.; 15 School of Pharmacy, University of Auckland; NZ.; 16 Montreal Neurological Institute, McGill University; Montreal, QC, Canada.; 17 Institute for Anatomy I, Medical Faculty and University Hospital Düsseldorf, Heinrich Heine University Düsseldorf; Düsseldorf, Germany.; 18 Institute of Neurosciences and Medicine (INM-1), Research Centre Jülich; Jülich, Germany.; 19 University of Cologne, Faculty of Medicine and University Hospital Cologne, Department of Child and Adolescent Psychiatry, Psychosomatics and Psychotherapy; Cologne, Germany.

**Keywords:** fMRI, MEG, functional connectivity, resting-state, brain network, connectome, PET, neurotransmitter, noradrenaline

## Abstract

The human brain is organized into interacting functional systems. Their underlying neurobiological mechanisms remain difficult to study in vivo^1,2^. Here, we adopt a topological framework to quantify the association between neurobiology and brain functional connectivity derived from both resting-state functional magnetic resonance imaging (rsfMRI) and magnetic encephalography (MEG). Across six healthy adult cohorts (n = 19–112), regional variation in rsfMRI connectivity robustly aligns with the distribution of neurotransmitter receptors and transporters. We find that low-frequency functional synchronization measured by rsfMRI is predominantly modulated by decreased regional availability of multiple receptors and transporters. These patterns are present in every single subject, replicate across all cohorts, and are mirrored in MEG, where high-frequency synchronization increases with availability of the same receptors and transporters. Most prominently, we observe noradrenergic modulation of functional connectivity in a sensorimotor-posterior-insular network that is consistently detected across individuals and is linked to autonomic arousal. In pharmacological and clinical samples, the associations are sensitive to manipulation of the respective neurotransmitter systems and are altered in patients with early psychosis, aligning with clinical symptomatology. These findings provide biological insight into typical and atypical functional organization of the human brain using a framework linking underlying neurobiology to the functional connectome (NEOFC).

Resting-state functional connectivity (rsFC)^3^ has become the primary framework for characterizing brain functional organization. Resting-state FC networks and, more recently, cortical gradients have been identified across various neuroimaging modalities and are now considered a core feature of functional brain architecture^4-7^. A central assumption in rsFC research is that the observed temporal synchrony between regions is driven by common neural activity^2^. However, despite three decades of study, the biological basis of rsFC – characterized through network, gradient, and related perspectives – remains only partly understood.

In humans, evidence linking macro-scale rsFC networks to specific cellular and molecular mechanisms is still largely indirect and often extrapolated from non-human animal studies^1,8^. Progress has been hindered by the limited neurobiological specificity of in vivo connectivity measures. Electro- and magnetoencephalography (E/MEG) capture aggregated neural activity, but their cellular and neurotransmitter origins remain difficult to disentangle^9^. In contrast to these more direct measures of neural activity, blood-oxygenation-level-dependent (BOLD) functional MRI reflects cumulative local metabolic demand shaped by neurovascular coupling and other non-neuronal physiological processes^1,2^. The limited information on underlying neurobiology provided by both neuroimaging modalities restricts the use of rsFC to investigate the pathophysiological mechanisms underlying functional variation across groups, and even more so across individuals. To address this constraint, we introduce a framework that captures the associations between individual rsFC and expected underlying biology. The revealed associations are robust, generalizable, related to other physiological processes, modifiable by intervention, and affected by clinical conditions, providing a new perspective for the study of macro-scale functional organization of the human brain.

In recent years, we and others have shown that the spatial topology of resting-state brain activity and connectivity derived from haemodynamic and electrophysiological data is associated with specific neurobiological features^10-23^. For example, the magnitude of blood flow changes induced by dopaminergic medication follows the expected distributions of dopaminergic receptors in the brain^10^. Pharmacological modulation of regional BOLD signals can be understood through the expected distributions of the respective drug targets^22,24^. BOLD alterations in neurological and neurodevelopmental disorders co-localize with affected neurotransmitter systems, and normative connectome organization is related to inter-regional similarity in receptor and transporter expression^13-17^.

The cross-modal co-localization approaches employed by prior studies largely focused on regional estimates of neural activity or connectivity, disregarding information on the brain’s network organization. However, their underlying assumption – if a receptor’s activity contributes to the measured neural signal, then modulating receptor activity should modulate the signal in brain regions with higher receptor availability – can be extended to functional connectivity. If a receptor is active across multiple brain regions contributing to the measured signal, its activity should introduce a characteristic pattern to the regional signals, which may result in increased synchronization between regions with high receptor abundance. Extending this logic, if a receptor supports transmission of local, short-range information, thereby increasing local signal entropy, then the receptor’s relative absence may facilitate interregional synchronization.

Building on these theoretical considerations, here we introduce a framework for evaluating the Neurobiologically Enriched Organization of Functional Connectivity (NEOFC). We quantify changes in rsFC as a function of regional receptor and transporter density estimates, obtained from nuclear imaging in healthy volunteers and spanning all major neurotransmitter systems^25,26^. A derived subject-level index aggregates the topological relationship between an individual rsFC connectome and specific neurobiological tissue properties. In multiple independent rsfMRI datasets, we show that these indices of neurobiology-dependent functional synchronization capture a robust and reliable component of brain network organization at both group and individual levels. Extended effects in MEG connectomes demonstrate that this network-organizational component generalizes across modalities. Close links between rsFC in a noradrenaline-rich sensorimotor-insular network and autonomic arousal support the neurobiological relevance of the framework. Sensitivity to pharmacological manipulation and to rsFC alterations in early psychosis further illustrate its clinical potential. All methods are openly available for the community to build upon.

## A framework linking typical neurobiological signals to individual connectomes

**Fig. 1:**
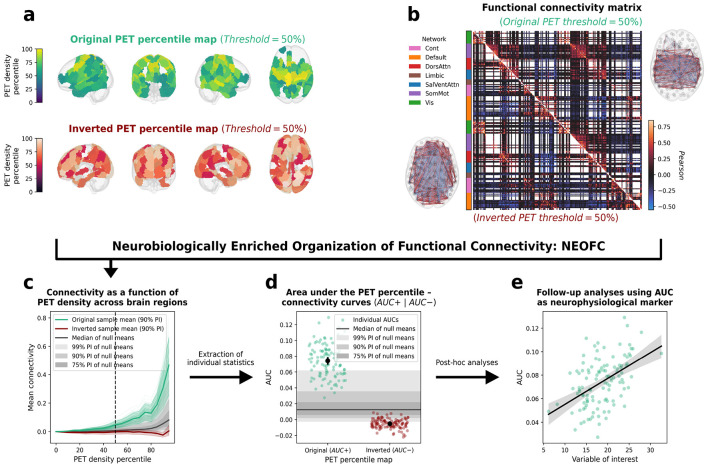
Neurobiologically enriched organization of functional connectivity (NEOFC) **a**: Cortical parcels (Schaefer200 parcellation, n = 200) color-coded by percentile rank of a given reference atlas (here: the noradrenaline transporter); percentile threshold 50% shown as an example. **b**: An rsFC matrix (here: group average correlation strength) thresholded by the 50th percentile of the reference atlas (top 50% densest parcels: top right; bottom 50%: bottom left); brain plots: thresholded connectomes projected on a standard glass brain. **c**: NEOFC curve: mean rsFC of included parcels as a function of the density percentile threshold, computed for the original reference map (green) and the spatially inverted reference map (dark red). Colored shaded area: group mean ± 90% PI across individuals. Grey shaded area: null distribution based on null models of the reference map. **d**: Individual AUC+ and AUC− scores, quantifying the area under the original and inverted NEOFC curves, respectively (each dot: one participant). **e**: Illustrative downstream application: AUC+ scores associated with a continuous variable of interest via linear regression (scatter: individual participants; line: regression fit). Abbreviations: rsFC: resting-state functional connectivity; PI: percentile interval; AUC: area under the curve.

The NEOFC framework (Fig. 1, Animation S1) quantifies the topological association between the spatial distribution of a neurobiological marker across brain regions (reference atlas; here, a group-average nuclear imaging map; Fig. 1a) and the functional network defined over the same regions (connectome; here, an individual’s 200-region-by-region Pearson rsFC matrix; Fig. 1b). We obtain this association by (i) converting the reference atlas to percentiles, (ii) masking the connectome according to atlas percentile thresholds ranging from 0 to 95, (iii) calculating the mean rsFC between regions remaining after each threshold step, and (iv) condensing the result in a curve of percentile thresholds versus mean rsFC (Fig. 1c). This relationship is then summarized as a single score by calculating the area under the curve (AUC) after subtracting the global connectivity (mean rsFC at percentile 0, i.e. of all connections; Fig. 1d). These AUC scores capture the overall association between each individual connectome and reference atlas and are used for all main and follow-up analyses (Fig. 1e). The AUC serves as the primary aggregate metric because it captures the overall connectome-reference relationship independent of curve shape. Using this approach, we test for positive associations, such that synchronization is stronger between regions with higher density of a given neurobiological atlas (AUC+). We then repeat the analysis using the spatial inverse of each neurotransmitter atlas to test for stronger synchronization between low-density regions (AUC−; Fig. 1a-d). To guard against spurious effects driven by shared spatial structure^27^, each association is evaluated against null reference atlases preserving spatial autocorrelation and interhemispheric symmetry^25^. This allows us to calculate non-parametric exact p values on group and individual levels, which we correct for the effective number of tests across atlases within each main analysis (p_Meff_). In sensitivity analyses, we evaluated both alternative brain parcellations and an alternative aggregation score focused on the quadratic-like shape of some percentile-rsFC curves (second-degree polynomial fit; Supplementary Methods).

We assembled a database of 25 reference atlases derived from independent healthy adult nuclear imaging studies^25,26^, using a harmonized registration procedure to ensure comparability and spanning all major neurotransmitter systems as well as metabolism, synaptic density, and transcriptomic activity (Tab. S1). For a positive control analysis, we additionally obtained probabilistic atlases of 12 established resting-state networks^28^. Individual connectomes were derived from rsfMRI and source-reconstructed MEG data from a subset of the Human Connectome Project Young Adult sample (HCP-YA)^29^, alongside rsfMRI data from one physiology study, three pharmacological studies, and one clinical case-control study (Tab. S2). The HCP-YA rsfMRI dataset served as the discovery sample for the main analyses (n = 112, 64 female), and findings were tested for replication in healthy, untreated, adult subsamples from the other five rsfMRI datasets.

### NEOFC is sensitive to canonical resting state network structure

**Fig. 2:**
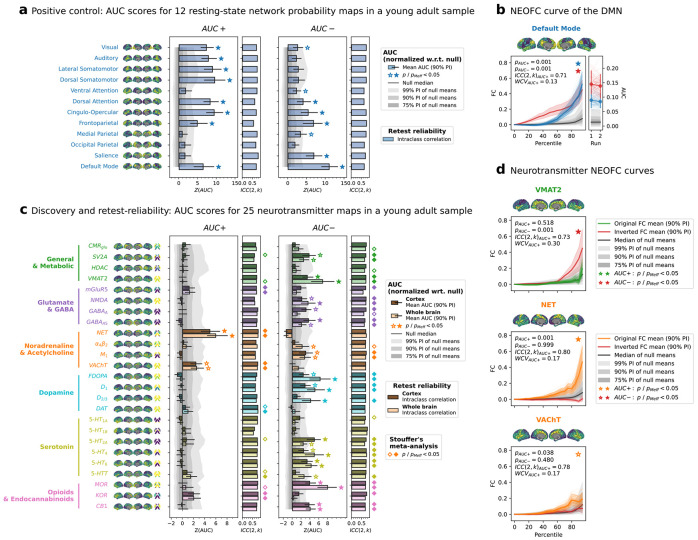
Resting-state fMRI functional connectivity organizes along neurobiology **a**: AUC+ and AUC− scores (z-scored against a null distribution of spatially matched reference maps) for 12 resting-state network atlases used as a positive control, together with test-retest reliability (ICC(2,k); Schaefer200 parcellation, first scanning run). Bars: group mean; error bars: 90% PI across individuals; grey shaded areas null distributions of the AUC group means, z-scored similarly to the observed scores for visualization. Brain maps show the parcellated reference atlases converted to percentiles. **b**: Exemplary NEOFC curve for the DMN probability atlas during the first scanning run (original percentile-FC curve: blue; inverted test: dark red; colored shaded area: 90% PI across individuals; grey shaded area: null distribution). Right side: comparison of AUC estimates from run 1 and 2 (thin lines: individual subjects; thick line, dots, and errorbars: group mean with 90% PI). **c**: AUC+ and AUC− scores for 25 nuclear imaging reference maps, displayed separately for Schaefer200 (darker bars) and Schaefer200 + 16 subcortical parcels (brighter bars); shown as in **a**. **d**: NEOFC curves for three selected monoaminergic transporter maps (VMAT2, NET, VAChT); displayed as in **b**. Abbreviations: AUC: area under the curve; ICC: intraclass correlation coefficient; WCV: within-subject coefficient of variability; PI: percentile interval; pMeff: p-value corrected for effective number of comparisons; DMN: default mode network; CMR_glu_: cerebral metabolic rate of glucose; SV2A: synaptic vesicle protein 2A; HDAC: histone deacetylase; VMAT2: vesicular monoamine transporter 2; mGluR5: metabotropic glutamate receptor 5; NMDA: N-methyl-D-aspartate receptor; GABA_A, A5_: gamma-aminobutyric acid type A, A5 receptor; NET: noradrenaline/norepinephrine transporter; α4β2: alpha-4 beta-2 nicotinic receptor; M_1_: muscarinic acetylcholine receptor M1; VAChT: vesicular acetylcholine transporter; FDOPA: fluorodopa; D_1, 2/3_: dopamine receptor 1, 2/3; DAT: dopamine transporter; 5-HT_1A, 1B, 2A, 4, 6_: serotonin receptor 1A, 1B, 2A, 4, 6; MOR/KOR: mu/kappa-opioid receptor; CB1: cannabinoid receptor type 1.

The canonical organization of the resting functional connectome is described by the well-established resting-state networks^2,4^. Probabilistic atlases map the probability of a region to belong to a given network, i.e., regions with high probability for a given network are likely to be connected to each other. In the NEOFC framework, such atlases should yield strong effects, acting as a positive control. In line with this, we found significant AUC+ effects for 8 out of 12 tested resting-state networks in the HCP-YA sample (p_Meff_ < 0.05; Figs. 2a/b; Tab. S2), indicating increased rsFC between regions with high probability of a given network. Interestingly, 5 networks, including 3 of the 4 which did not show AUC+ effects, also showed significant AUC− effects, indicating stronger synchronization between regions with low network probability and likely reflecting the known spatial anticorrelation of some of the networks ((p_Meff_ < 0.05; Fig. 2a/b). This pattern was generally robust to the alternative parcellation schemes and aggregation metric (Fig. S1; Tab. S3).

### The fMRI resting-state connectome is robustly associated with neurobiological signals

Having established that NEOFC can capture known topological relationships, we next tested for the neurobiological signals underlying rsFC organization in the HCP-YA fMRI dataset. We observed the strongest AUC+ effect for the noradrenaline transporter (NET; p_Meff_ < 0.05; Fig. 2c/d; Fig. S2; Tab. S4), arising from higher connectivity between regions with stronger NET signals. The effect was significant in each of 112 discovery subjects (p < 0.05; Fig. S3) and in all five replication samples (p_Meff_ < 0.05; Fig. S4). A meta-analysis across all six healthy datasets, suited to also detect weaker effects, additionally revealed significant AUC+ effects of acetylcholine, dopamine, and serotonin transporters (VAChT, DAT, 5-HTT) as well as glutamate and opioid receptors (mGluR5, KOR; p_Meff_ < 0.05; Fig. 2c; Fig. S5; Tab. S5). In contrast to the few AUC+ effects, AUC− effects were observed across all studied neurotransmitter systems (p_Meff_ < 0.05; Fig. 2c/d; Tab. S4). This pattern of results was supported by analysis of the delta between AUC+ and AUC− effects, which should diminish influences of shared spatial patterns not captured by the null models (Fig. S6; Tab. S4). The main effects were robust to different parcellation schemes, removal of interhemispheric connections, as well as the alternative aggregation metric (Supplementary Results; Fig. S6/7; Tab. S4).

### Neurobiological rsFC organization is reliable and reproducible

**Fig. 3:**
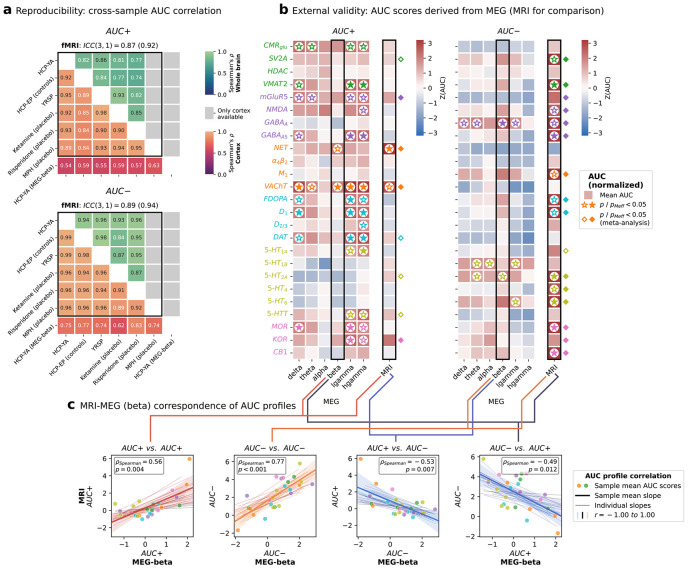
Neurobiological rsFC organization is reproducible and extends to MEG **a**: Pairwise Spearman's rho between mean AUC+ (top) and AUC− (bottom) profiles across 6 fMRI cohorts and one MEG cohort (25 nuclear imaging reference maps; Schaefer200 and Schaefer200 + subcortex parcellation). Lower triangle: cortex; upper triangle: whole-brain. ICC(3,1) reported for fMRI cohorts (first value: cortex; value in brackets: whole-brain). **b**: AUC+ and AUC− scores for 25 nuclear imaging reference maps derived from MEG AEC FC matrices, displayed as a heatmap (Schaefer200, z-scored against null distribution); right column: fMRI results for reference. **c**: Correspondence between fMRI and MEG-beta AUC profiles (z-scored against null) across 25 reference maps (each dot: sample-mean AUC value of one reference atlas; thick line: corresponding regression fit with 95% bootstrapped CI; thin lines: individual regression fits without single values; color: Spearman's rho). Four combinations shown: fMRI AUC+ vs. MEG AUC+, fMRI AUC− vs. MEG AUC−, fMRI AUC+ vs. MEG AUC−, fMRI AUC− vs. MEG AUC+. Abbreviations: AUC: area under the curve; fMRI: functional magnetic resonance imaging; MEG: magnetoencephalography; ICC: intraclass correlation coefficient; FC: functional connectivity; AEC: amplitude envelope correlation; CI: confidence interval; pMeff: p-value corrected for effective number of comparisons.

One-day test-retest data from the HCP discovery dataset served to evaluate the reliability of the NEOFC framework. All significant AUC outcomes showed a good to excellent retest reliability, supporting robustness of the respective findings [ICC(2,k) > 0.7; within-subject coefficient of variation < 0.25; Fig. 2a; Tab. S6]. Comparison of the AUC+ and AUC− profiles between six independent datasets showed very good to excellent reproducibility (Fig. 3a; Fig. S4; Tabs. S7/8).

### Electrophysiology validates and enhances hemodynamic findings

Electrophysiological measures are conceptually more closely related to neuronal activity than the hemodynamic signals measured by rsfMRI and thus should contain more fine-grained information on underlying neurobiology. We extended our analysis of the HCP-YA dataset by studying source-reconstructed resting-state MEG connectomes across six frequency bands (amplitude envelope correlations; n = 33, 16 female)^21,30^. Compared to rsfMRI, AUC profiles derived from MEG showed a shift towards higher sensitivity to AUC+ effects and reduced sensitivity to AUC− effects (Fig. 3b; Fig. S8; Tab. S9). Most AUC+ effects were observed in low- and high-gamma frequency bands, spanning several neurotransmitter systems, while the beta band was dominated by NET and VAChT AUC+ effects. In contrast to rsfMRI, AUC− results largely did not reach significance levels with the notable exception of GABA_A_ (p_Meff_ < 0.05).

Group- and single-subject level AUC+ and AUC− profiles displayed strong similarity between MEG and rsfMRI, with the strongest alignment observed for the MEG beta band (Fig. 3c; Fig. S9; Tab. S10). The results remained stable with different parcellation resolutions (Figs. S10a/b; Tab. S9). Analyses of individual AUC estimates across subjects per neurotransmitter map indicated a limited association between both modalities (Fig. S11; Tab. S11).

### Region-specific influences on neurobiological rsFC organization

Next, we assessed how the general connectome-neurobiology associations captured by the AUC indices were influenced by rsFC patterns of individual brain regions. We employed a leave-one-out approach identifying regions/connections whose exclusion most strongly influenced the aggregated AUC metric as identified in the meta-analytic approach^31^. Second, we evaluated AUC scores calculated at systematically lowered upper percentile bounds to determine if the observed effects were driven by only a few regions with extreme expression or a continuous topological association across a larger subnetwork.

**Fig. 4:**
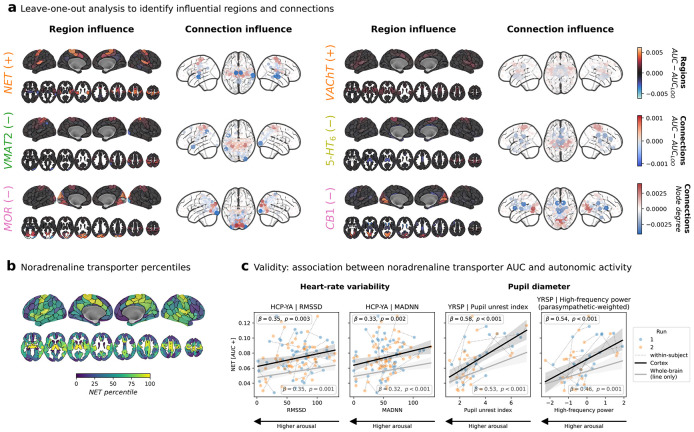
Neurobiological rsFC organization is driven by regional subnetworks and reveals a sensorimotor-insular component related to autonomic physiology **a**: Regional (left, leave-one-region-out) and connection (right, leave-one-connection-out) influence on AUC for 6 selected reference maps and directions: NET (AUC+), VAChT (AUC+), VMAT2 (AUC−), 5-HT6A (AUC−), MOR (AUC−), and CB1 (AUC−) (Schaefer200 + subcortex parcellation). Color indicates direction and magnitude of influence. **b**: NET density percentile map projected onto the Schaefer200 + subcortex parcellation, shown for spatial reference. **c**: Association between NET AUC+ and autonomic physiological measures across two cohorts: HRV (RMSSD and MadNN; HCP-YA) and pupil-based arousal indices (PUI and HF-P power; YRSP). Scatter: individual observations, repeated measures are connected; black: Schaefer200 parcellation; grey: Schaefer200 + subcortex parcellation; line with shaded area: regression fit across all data with 95% bootstrapped CI. LMM used for statistical inference, controlling for mean FD, global connectivity, and demographic covariates. Abbreviations: rsFC: resting-state functional connectivity; AUC: area under the curve; HRV: heart rate variability; RMSSD: root mean square of successive differences; MadNN: median absolute deviation of NN intervals; PUI: pupil unrest index; HF-P: high-frequency power of the arousal-related pupil signal range (0.25–0.50 Hz); LMM: linear mixed model; FD: framewise displacement; CI: confidence interval; see Fig. 2 for reference map abbreviations.

Both, AUC+ and AUC− displayed distinct topological associations (Figs. 4a and S12a/b). We found the NET AUC+ pattern to be most strongly influenced by posterior insula, lateral and medial sensorimotor, and supplementary motor areas, regions with notably high levels of NET (Figs. 4a and S12a). We furthermore found that the NET AUC+ effect emerged even when considering only the lowest 40% of the regional NET distribution. This indicates a widespread topological brain network association, peaking in specific high-NET regions. AUC− scores derived from serotonergic and opioidergic atlases displayed even more extended patterns (Fig. S13; Tab. S12).

So far, we introduced the NEOFC framework as a method to study topological associations between human brain connectome organization and neurobiological signals. The framework rests on the assumption that the spatial pattern exhibited by a neurobiological reference atlas is reflected in individual brain network topology. From the extent of this cross-modal spatial association, we derived interpretable markers of individual connectome-neurobiology relationships. These markers proved robust, reliable, and reproducible in rsfMRI data, but are also – or even more so – present in electrophysiological data, supporting a neuronal origin. In the following, we will investigate the (patho-)physiological relevance of our approach by exploring the notable noradrenergic effect, demonstrating that derived indices are sensitive to pharmacological modulation, and conducting a clinical application experiment.

### A noradrenergic motor-insular component reflects autonomic arousal

AUC+ associations with NET were the strongest and most consistent signal detected in the above analyses. We next tested for their neurobiological relevance, thereby providing external evidence supporting the NEOFC framework. The NET is typically located presynaptically at cortical and subcortical projection sites of noradrenergic neurons^32^, a subpopulation of which form the locus coeruleus (LC). Accordingly, the NET reference atlas showed the highest density in lateral and medial sensorimotor cortices, insula, and medial subcortical regions (Fig. 4b), the cortical regions of which strongly influenced the AUC+ effects.

On the basis of this anatomical pattern, the well-established autonomic functions of the LC^33^, the consistent NET effect across subjects and samples, and the observed regional importance profile, we hypothesized that the signal reflects a general neurobiological process related to autonomic arousal. Considering this hypothesis, the similarity of the NET atlas to the sensorimotor-association axis, and prior reports on a global fMRI component associated to peripheral physiology^34^, we first confirmed that this association persisted after spatial regression of several atlases capturing brain vasculature as well as functional and microstructural organization (Fig. S14; Tab. S13). To minimize the risk of physiological artifacts, we further implemented a strict 36-parameter confound regression scheme during rsfMRI preprocessing^35^.

Across two independent datasets employing different measurements of peripheral autonomic activity, NET AUC+ scores were robustly associated with autonomic arousal measures across subjects and sessions (Fig. 4c; Tab. S14). First, NET AUC+ was significantly associated with in-scanner heart rate variability in the HCP-YA subsample (n = 64, 103 observations; Fig. S15). Second, it was strongly associated with low-frequency pupil diameter fluctuations in the Yale Resting State fMRI/pupillometry sample^36^ (YRSP; n = 25, 49 observations; Tab. S2; Fig. S16). Both analyses indicate an inverse relationship between NET AUC+ and autonomic arousal, with higher arousal linked to lower synchronization between regions with high NET availability, an association possibly related to the reported interruption of functional networks following tonic LC activation^37^.

### Neurobiological rsFC organization is sensitive to pharmacological modulation

As demonstrated above, the NEOFC framework is sensitive to neurobiological properties that underlie brain functional network organization. NEOFC metrics should therefore be altered by interventions affecting the same neurobiological systems. We tested this hypothesis in three healthy adult datasets (Tab. S2) applying four psychotropic substances – methylphenidate (MPH), risperidone, ketamine, and midazolam – in placebo-controlled, within-subject, crossover trials^24,38,39^. We systematically evaluated drug effects for both AUC+ and AUC− scores calculated on subject-/session-level across all reference atlases in separate mixed linear models (controlled for mean motion and global connectivity).

**Fig. 5:**
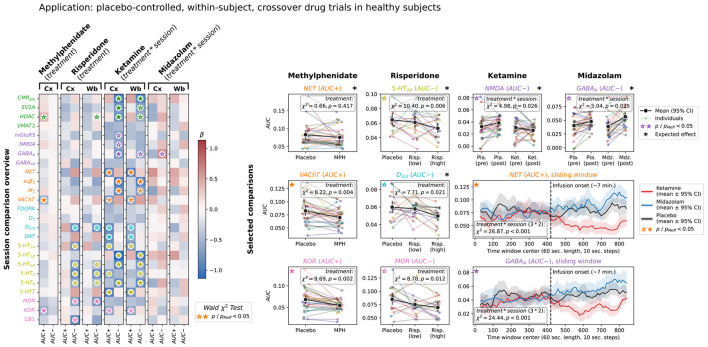
Neurobiological rsFC organization is modulated by pharmacological agents Left, session comparison overview: LMM beta coefficients for treatment effects on AUC+ and AUC− across 25 nuclear imaging reference maps and 4 drug challenges for Schaefer200 and Schaefer200 + subcortex parcellations (methylphenidate and risperidone: treatment effect; ketamine and midazolam: treatment × session interaction). Color: standardized beta coefficient; significance markers indicate Wald χ^2^ test p values. Right, selected comparisons, all for Schaefer200: small panels: individual AUC values per session for expected and strongest target effects (expected targets marked with an asterisk; methylphenidate: NET, VAChT, KOR; risperidone: 5-HT_2A_, D_2/3_, MOR; ketamine: NMDA; midazolam: GABA_A_). Colored dots with thin lines: individual subjects; Black dots with error bars: group mean with 95% bootstrapped CI. Lower right big panels: sliding window timecourses of mean AUC+ (NET) and AUC− (GABA_A_) across the infusion session (60-second windows, 10-second steps); ketamine: red; midazolam: blue; placebo: black; shaded area: 95% bootstrapped CI. Abbreviations: LMM: linear mixed model; AUC: area under the curve; FC: functional connectivity; CI: confidence interval; pMeff: p-value corrected for effective number of comparisons; see Fig. 2 for reference map abbreviations.

A combined overview of the results is shown in Fig. 5 (Tab. S15). First, MPH is expected to affect noradrenaline and dopamine transporters. We could not confirm effects of MPH on NET or DAT AUC in the available dataset, but we observed negative associations with AUC+ scores for an acetylcholine transporter (VAChT) and opioid receptor (KOR), indicating reduced rsFC between high-density brain regions under MPH influence. Risperidone, in contrast, showed effects well in line with its known dopaminergic and serotonergic receptor targets. The strongest effects of risperidone were a reduction of AUC− scores for the 5-HT_2A_ and D_2/3_ receptors compared to placebo. For ketamine, we observed widely distributed effects on AUC+ and AUC−. While the main target of ketamine, the NMDA receptor, indeed showed a significant reduction of AUC− scores, we observed many stronger effects, the strongest being a reduction of NET AUC+. Lastly, exposure to midazolam resulted in an increase in AUC− of the expected target receptor, GABA_A_, as the only significant effect. We then tested for potentially opposing effects of ketamine and midazolam in a 3-factor model. The strongest effects following a “midazolam > placebo > ketamine” pattern were observed for NET AUC+, GABA_A_ AUC−, and serotonergic AUC− scores.

As ketamine and midazolam were applied intravenously during fMRI scanning, the data allowed us to further explore the acute pharmacological modulation of NEOFC by running a sliding-window analysis. For both drugs, we found a strong deviation of time-resolved AUCs from both placebo and pre-infusion data (Fig. 5, lower right), confirming the modulatory drug effects and the ability of the method to track rsFC patterns dynamically over time.

### Neurobiological rsFC organization is sensitive to network alterations in early psychosis

The pharmacological analyses indicate that the NEOFC framework carries potential for studying pathophysiological processes involved in psychiatric and neurological disorders. We explored this in a clinical experiment in the HCP Early Psychosis dataset (HCP-EP; n = 96 patients, n = 55 controls; Tab. S2). We systematically tested for group differences in both AUC+ and AUC− between the full patient and control cohorts, as well as after stratifying the psychosis group based on medication status (current and lifetime exposure; group differences in covariates: Tab. S16). Finally, we calculated partial Spearman associations of AUC indices with symptom and general cognitive function scales as well as medication intake in the psychosis group.

**Fig. 6:**
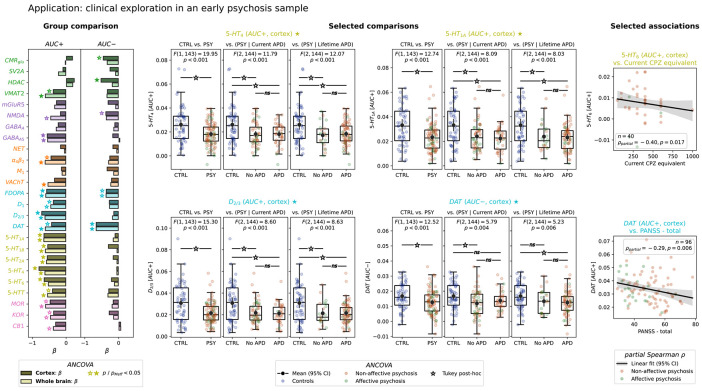
Neurobiological rsFC organization is altered in early psychosis Left, group comparison: type II ANCOVA beta coefficients for CTRL vs. PSY group differences in AUC+ and AUC− across 25 nuclear imaging reference maps (site-harmonized data; covariates: age, sex, mean FD, global connectivity, current antipsychotic dose). Darker bars: Schaefer200; brighter bars: Schaefer200 + subcortex; significance markers indicate pMeff. Middle, selected comparisons: individual AUC values per diagnostic group for the 4 strongest effects (5-HT_4_, 5-HT_1A_, D_2/3_: AUC+; DAT: AUC−). Each panel shows three diagnostic contrasts: CTRL vs. PSY, CTRL vs. PSY stratified by current antipsychotic dose, and CTRL vs. PSY stratified by lifetime antipsychotic use. Subjects are additionally color-coded by controls (blue), and non-affective (orange) vs. affective psychosis (green). Dots: individual subjects; boxplots: median, quartiles and 1.5*interquartile range; black dots with errorbars: mean and 95% bootstrapped CI; brackets with significance markers: Tukey HSD post-hoc comparisons. Right, selected associations: Correlations between AUC+ and clinical variables within the PSY group (scatter: individual subjects; line: regression fit with 95% bootstrapped CI; partial Spearman’s rho reported). Abbreviations: AUC: area under the curve; FC: functional connectivity; ANCOVA: analysis of covariance; CTRL: healthy controls; PSY: psychosis; FD: framewise displacement; pMeff: p-value corrected for effective number of comparisons; see Fig. 2 for reference map abbreviations.

We found a strong reduction of primarily AUC+ scores in psychosis across various neurotransmitter systems (Fig. 6, left; Tab. S17). Serotonergic, followed by dopaminergic indices showed the strongest significant reductions (Fig. 6, center). These effects indicate reduced rsFC between serotonin and dopamine receptor-rich brain areas in psychosis. Notably, the serotonergic 5-HT6 AUC+ correlated negatively with current antipsychotics intake (Fig. 6, right). For the dopamine transporter, we found significant reductions for both AUC+ and AUC− out of which AUC+ reduction was associated with higher symptom scores (Fig. 6, right). More generally, correlation patterns with clinical scales and medication intake were strongest with serotonergic and dopaminergic AUC+ indices, however, none survived correction for multiple comparisons (Fig. S17; Tab. S18). Medicated and unmedicated psychosis subgroups did not differ. The effects were largely similar when subcortical parcels were included (Fig. 6; Tab. S17).

## Discussion

Here we examined how functional synchronization of the human brain relates to underlying neurobiology using an integrative framework based on brain network topology. We showed that functional connectivity measured with both haemodynamic and electrophysiological imaging modalities is robustly associated with neurotransmission, with stronger connectivity observed within networks characterized by high or low expression of specific receptors or transporters. These effects were highly reliable and reproducible at group and individual levels. The close association between noradrenergic AUC scores and measures of physiological arousal provides evidence for the neurobiological relevance of the observed effects. The AUC scores were modulated by pharmacological interventions and altered in patients with psychotic symptoms, highlighting their potential for clinical research, which we facilitate by providing the underlying method as a Python package (mapconn).

We first validated the NEOFC approach using established resting-state networks as a positive control. For eight of the twelve networks, we observed the expected synchronization effects, indicating high sensitivity of the method to interregional rsFC organization. When applied to a comprehensive database of neurotransmission atlases, most of these showed topological associations in terms of increased rsFC between regions with high or low density of the respective receptors or transporters. The strongest effects were consistently observed across all evaluated subjects and cohorts in both rsfMRI and MEG data, pointing to a general neurobiological basis. Individual AUCs, as well as group-level and single-subject AUC profiles across reference atlases, displayed good to excellent test-retest reliability.

The strongest evidence for a neurobiological source of the observed AUC effects comes from MEG, where AUC+ effects of NET and VAChT emerged in the beta band. As a more direct measure of neural activity, MEG is less likely to be biased by physiological confounds that are typically associated with rsfMRI, i.e., peripheral physiological signals. With the exception of GABA_A_, only AUC+ associations passed stringent significance thresholds, extending the recently reported neurotransmitter-related modulation of local intracranial EEG spectra to rsFC^40^. In line with the known association between gamma oscillation power and aggregated neural firing rates^41,42^, we observed the strongest MEG-neurobiology associations in gamma frequency bands. While gamma-band activity is typically generated locally, long-range network organization is enabled via cross-frequency coupling^43^, whereby fast local gamma activity is modulated by slower rhythms^41,44^.

With rsfMRI, we observe only a few AUC+ associations. The most consistent effects were found for noradrenaline, followed by acetylcholine transporter availability. The noradrenaline effects were primarily driven by a sensorimotor-insular network, overlapping with known interoceptive and sensory-integrative brain networks^45^. Two independent datasets provide convergent evidence for an association between these noradrenaline effects and arousal, indexed by heart rate variability and pupil unrest. The tonic noradrenaline release by the LC is thought to interrupt the activity of functional networks to promote rapid behavioral adaptation^37^. This process reconfigures network activity from a tightly coupled, low-arousal state toward cross-network integration^37,46^. Consistent with this mechanism of action, we find higher arousal to be associated with lower individual AUC+ scores. More generally, a meta-analysis across all rsfMRI cohorts revealed a tendency of all neurotransmitter transporters towards AUC+ effects. Transporters are mainly presynaptic and closely tied to long-range ascending projection systems, so their network signatures may be sufficiently strong and spatially coherent to be detected in hemodynamic rsFC^33,47^.

The majority of rsfMRI effects displayed increased rsFC between regions with low density of the neurobiological atlases as indicated by AUC− effects. Notably, these effects were largely absent in MEG data. Such associations may be expected for receptors or transporters carrying primarily local information, i.e. if a receptor’s activity rendered the local BOLD signal more unique, its absence could facilitate synchronization. A more integrative explanation may lie in frequency specific modes of interregional communication^48^. Regions interact through neurotransmission often at higher frequencies, such that synchronization is found in fast dynamics and therefore captured by MEG (gamma)^41-43^. In contrast, the same neural sources are observed in rsfMRI through neurovascular coupling and after heavy temporal filtering, which can attenuate interregional information and leave a more local-appearing metabolic covariance pattern^1,49^. Only ultra-slow fluctuations in neural population activity, producing large-scale networks with high spatial coherence, are captured by rsfMRI. Such signals may be tracked especially by MEG beta band amplitude envelopes. The beta band showed the strongest rsfMRI-MEG cross-modal convergence in our study and is generally considered spatiotemporally robust with a favorable signal-to-noise ratio^5,50,51,30^. Strongly supporting this reasoning, Sastre-Yagüe et al.^52^ recently demonstrated that causal manipulation of cortical excitability in mice induced opposing effects on high frequency spectra, as captured by MEG, versus ultralow frequency spectra, as captured by rsfMRI. In line with this notion of opposing effects, we find a negative association between AUC− profiles observed with rsfMRI and AUC+ profiles observed with MEG and vice versa. Despite clear dissociations in the individual significant AUC effects, significant cross-modal correlations between AUC+ and AUC− profiles support the notion that both imaging modalities actually capture information from high and ultra-slow frequency spectra, though clearly with preferential sensitivity.

Using pharmacological challenge data, we tested whether the AUC scores are modifiable by targeted interventions. Consistent with their mechanisms of action, we found that risperidone, ketamine, and midazolam modulated the AUC of receptors with high affinity of the respective compounds^53-56^. Risperidone reduced AUC− estimates of multiple serotonergic and the dopamine D_2/3_ receptors, which may be expected from its receptor antagonism, with downregulation reducing the desynchronization. Ketamine, an NMDA receptor antagonist, is known for its widespread direct and downstream effects on a multitude of neurotransmitter signaling pathways^55,57,58^. Consistently, we found non-specific ketamine effects on AUC across most of the evaluated neurotransmitter systems. AUC effects of ketamine and midazolam displayed opposing patterns for GABA_A_ AUC− and NET AUC+. While ketamine generally increases sympathetic drive, a property also linked to its antidepressant effects^59^, midazolam generally shows inhibiting effects. Despite its GABA-mediated inhibitory properties and in contrast to the other compounds, midazolam generally increased rsFC, supporting the notion that deactivation rather than activation of a receptor may often support long-range rsfMRI network synchronization. Contrary to its mechanism of action as a noradrenaline and dopamine reuptake inhibitor, MPH showed AUC+ reductions only for a cholinergic transporter and the κ-opioid receptor. The limited rsfMRI sensitivity to its primary mechanism of action is consistent with previous studies reporting similar negative MPH findings for local activity measures^10^. While further investigation is necessary, possible explanations include its indirect mechanism of action as a reuptake inhibitor, direct or downstream effects of MPH on the opioid system^60^, and limited effects of MPH in healthy adults^61^.

AUC profiles in patients with psychotic symptoms differed from controls across most neurobiological systems, with the strongest alterations seen for dopaminergic and serotonergic neurotransmission, consistent with their established role in psychosis^62,63^. All of the observed effects indicate AUC reductions, with stronger effects for AUC+. This pattern, which was independent of antipsychotic medication and global connectivity in our early psychosis sample, suggests a general disruption of the typical coupling between neurobiology and large-scale rsFC organization in psychosis. The fact that the respective AUC+ scores were significant only in MEG but not in rsfMRI in control subjects alone raises interesting questions regarding the directionality of such a disruption as well as on the potential relevance of high frequency spectra for the observed findings. Future research is needed for corroboration of these findings, ideally with PET and electrophysiological imaging.

While all of these findings provide compelling evidence for the neurobiological relevance of NEOFC-derived measures, key limitations of the framework include (i) correlative nature of the non-interventional findings limiting causal interpretation, (ii) dependence on the quality and representativeness of PET-derived reference maps, (iii) reduced specificity due to intercorrelated maps, and (iv) current restriction to univariate analyses^64-66^. Validation was further limited by heart rate estimation based on pulse-oximetry (relative to electrocardiography) in HCP-YA and small, demographically narrow pharmacological cohorts applying drugs with partly broad binding profiles. Future work should focus on multivariate modeling across reference atlases, systematic comparison of connectivity estimators and metrics, integration with simultaneous fMRI-EEG and fMRI-PET, and clinical out-of-sample prediction. The framework is readily extensible to other biological priors, electrophysiological connectomes (e.g., EEG), task-based or dynamic rsFC, and within-subject mapping (e.g., structure–function coupling).

## Method

### Data sources and general processing

#### Ethics statement

No new data were acquired for this study. Ethical approval for the use of publicly available and restricted-access datasets including human demographic, behavioral, and neuroimaging data has been granted by the Heinrich-Heine-University, Düsseldorf, Germany. For information on ethical approvals for each dataset as provided by the local committees, we refer to the original sources cited in the text. Informed consent was obtained from each participant and their parents in the case of underage subjects (the latter applying only to a small subset of the HCP Early Psychosis sample).

#### Software

For connectomes which were not obtained directly from other publications, preprocessing of rsfMRI data was performed using the HCP pipeline^67^ for the HCP-EP sample and with FreeSurfer^68^ (7.4.1) and fMRIprep^69^ (24.0.1) for the remaining datasets. Postprocessing and extraction of connectomes was done with XCP-D^70^. All further analyses were performed in a Python (3.10.15) environment, relying on the NiSpace^25^ (0.0.1-beta.4) toolbox and mapconn (0.0.1) packages, as well as on routines from numpy^71^ (1.26.4), pandas^72^ (2.2.3), scipy^73^ (1.12.0), statsmodels^74^ (0.14.4), pingouin^75^ (0.5.5), neuroHarmonize^76^ (2.4.5), Nilearn^77^ (0.10.4), neuromaps^26^ (0.0.5), NeuroKit2^78^ (0.2.11), matplotlib^79^ (3.9.2), and seaborn^80^ (0.13.2).

#### Reference atlases

All deployed reference atlases, i.e., nuclear imaging, resting-state network probability, and covariate maps, were obtained directly through NiSpace in parcellated, tabular format. Originally, these data stem from independent studies conducted in healthy adults, which then shared their group-level outputs for public use. Sources of the nuclear imaging reference atlases are listed in Table S1^10,11,17,26,81-114^. A known problem with the currently available collections of nuclear imaging atlases is the heterogeneity of spatial registration target spaces, which are often unknown. To approach this, we registered all group-average maps to 2 mm MNI152NLin6Asym space using a non-linear deep-learning based method (SynthMorph v4)^115^. The resulting maps were masked with a liberal grey matter mask generated from the Harvard-Oxford atlas and scaled from 1e-6 to 1. Resting-state network probability maps, i.e., the probability that a given voxel belongs to a given canonical resting-state network across multiple subjects, were obtained from Dworetsky et al.^28^ and registered to MNI152NLin6Asym space as described above. Atlases used as spatial covariates were provided by several separate publications: T1/T2^29^, sensory-association axis^116^, cytoarchitectural gradient maps derived from post-mortem human histology^117,118^, as well as probability maps of gray matter and cerebrospinal fluid^119^, cerebral veins^120^, and arteries^121^.

#### Brain parcellations

We employed the Schaefer parcellation^122^ at 200-parcel density with and without 16 FreeSurfer subcortical parcels (“aseg”, excluding the brainstem) as the main parcellation scheme. To evaluate effects of different parcellation densities, we additionally tested 100- and 400-parcel densities with and without subcortical parcels only in the rsfMRI discovery and retest, MEG validation, and rsfMRI-MEG association analyses. The parcellations were applied directly in the target image spaces, i.e. in MNI152NLin6Asym for volumetric reference data, fsaverage for reference data originally provided in surface format, and fsLR for functional data.

#### Study samples and subject exclusions

Full sample descriptions and demographic details are provided in Supplementary Methods and Table S2. All rsfMRI sessions with mean framewise displacement (FD) > 0.3 mm were excluded (except the MPH dataset, for which preprocessed connectomes were obtained directly from the original authors). Briefly, the primary discovery sample was drawn from the HCP-YA dataset^29^ (n = 112, two resting-state runs; MEG data available for a subset of n = 33). The YRSP dataset^36,123^ provided simultaneous rsfMRI and pupillometry recordings from n = 25 subjects (49 sessions) for the physiological association analyses. Three placebo-controlled, randomized, within-subject crossover studies were included for pharmacological analyses: an oral MPH study^24^ (placebo vs. MPH; n = 30), an oral risperidone study^38^ (placebo vs. low vs. high dose; n = 17–19 sessions per condition), and an intravenous ketamine/midazolam study^39^ with pre- and post-infusion rsfMRI (placebo vs. ketamine vs. midazolam; n = 24–28 sessions per condition). The HCP-EP dataset^124^ served as the clinical application case, comprising 151 subjects across 4 acquisition sites (n = 96 early psychosis, n = 55 controls).

#### Resting-state fMRI processing

Full preprocessing details are provided in the Supplementary Methods. Briefly. the HCP-YA and HCP-EP datasets were obtained in “minimally preprocessed” format as generated by the HCP-pipeline^67,125^; all remaining datasets (except MPH, for which connectomes were provided directly^24^) were preprocessed using FreeSurfer and fMRIprep. All data was registered to the fsLR surface space (91k density). Except for MPH, postprocessing and connectome extraction was performed with XCP-D, applying a 36-parameter nuisance regression strategy with despiking and bandpass filtering (0.01–0.08 Hz)^35^. Pearson FC matrices were computed between parcellated time-series and Fisher z-transformed. For HCP-YA and HCP-EP, connectomes were averaged across phase-encoding directions within each run.

#### MEG processing

Source-reconstructed and parcellated resting-state MEG connectomes were provided by Shafiei et al.^30^; the processing was described in detail in the cited paper. Preprocessing and connectome extraction followed Brainstorm^126^ recommendations and included cleaning of the sensor-level data, reconstruction of source-level data on the individual fsLR surfaces, parcellation of the vertex-level time series, and connectome calculation using amplitude envelope correlation^127^ at 6 canonical electrophysiological bands (delta: 2–4 Hz, theta: 5–7 Hz, alpha: 8–12 Hz, beta: 15–29 Hz, low gamma: 30–59 Hz, and high gamma: 60–90 Hz). As we were interested in the shared signal across regions, we did not perform signal orthogonalization, preserving the original network topology. We test for statistical significance with spatial null models of the reference atlases while the individual input connectomes remain unchanged during the null runs. Therefore, any spurious signals that may bias the individual connectomes, e.g., arising from MEG signal leakage, will equally influence both observed and null runs, providing an exact control for false positives.

### Linking reference atlases to connectomes

#### Implementation

To derive the association between a parcellated atlas and an individual connectome (i.e., a region-to-region FC matrix), we (i) converted the reference atlas to percentiles of its own distribution, (ii) masked the connectome to keep only connections between regions that exceeded a specified reference atlas percentile, (iii) systematically repeated this for all percentiles in {0, 5, 10, …, 95}, and (iv) calculated the average FC of the masked connectome for each percentile, resulting in (v) a curve showing average FC as a function of percentile threshold. Global connectivity (the value at the 0th percentile, i.e., average FC across all connections) was subtracted from each curve to account for interindividual, acquisition- and processing-related differences in global connectivity. The area under the curve (AUC), computed via the trapezoidal rule, served as the prime aggregative index of the map–connectome association per individual. The inverse test (i.e., producing AUC− scores) was implemented by inverting each original reference atlas map around its mean, such that the highest-ranked regions became the lowest-ranked, and recomputing the steps lined out above.

We conducted several sensitivity analyses reported at appropriate places in the main text. In the initial rsfMRI analyses, we evaluated the leading coefficient of a second-degree polynomial fitted to the FC-percentile curve as an alternative aggregate metric, focussing on the shape of the curve rather than on the overall effect beyond the global connectivity. In the rsfMRI discovery analyses, to additionally control for spatial autocorrelation, we calculated the delta between aggregate outcomes from the original and inverted tests for further evaluation only. In a sensitivity analysis to assess if observed effects on rsFC were driven by the highest-density atlas regions, we systematically varied the maximum percentile threshold in AUC calculation from 5 to 90 and reported the lowest threshold above which significance emerged continuously. To test if the NET AUC+ effect was driven by possibly confounding spatial factors, we individually regressed each of a set of covariate maps from the NET atlas and reran the AUC+ estimation on the residuals.

#### Null models

Spatial patterns that are shared across brain-wide signals, but convey no information of interest, can inflate associations measures between the same signals^27^. Therefore, we evaluated each association against null models of the reference atlases, retaining spatial autocorrelation and interhemispheric symmetry. Null models were generated independently for parcellated cortical and subcortical data before conversion to percentiles. For cortical data, we employed a symmetric “spin test” on the fsLR spherical projection with nearest-neighbor reassignment between original and rotated coordinates^26,128^. As this process is only applicable to cortical data, null models for subcortical data were generated independently using Moran Spectral Randomization^129,130^ based on a Euclidean distance matrix. Interhemispheric symmetry was ensured through centerline-mirrored rotations in the first, and a near-symmetric distance matrix in the second case. We generated 1,000 null maps for each input reference map and recalculated NEOFC metrics with each. Exact *p* values were determined based on the resulting null distribution on individual as well as on group levels (group level: sample mean of observed scores vs. sample mean of null scores ).

To compare the derived scores akin to effect sizes while respecting non-normal null distributions, we calculated robust z-scores by subtracting the median of the null distribution from the observed statistic and dividing by the median absolute deviation scaled by 1.4826 (the consistency factor for a normal distribution). These z-scores were used to present NEOFC metrics across multiple reference atlases (i.e., Fig. 2a/b and 3b) and in all analyses that compared reference atlas profiles between cohorts or modalities (e.g., Fig. 3). This procedure minimized the influence of unwanted spatial influences on profile-level associations in which, e.g., different levels of autocorrelation across atlases could drive the results. Supplementary tables contain both original and z-scored values.

#### Multiple comparison correction

We empirically adjusted for alpha-error accumulation within sets of tests within each analysis by calculating the effective number of tests (M_eff_) based on eigenvalues of the Pearson correlation matrix of the input data to each analysis^131^. We then used the estimated factor to correct p values with Šidák’s correction as: p_Meff_ = 1 – (1 – p)^Meff^. This approach was applied to each analysis as described in the respective sections.

#### Positive-control and discovery analysis

The NEOFC framework was first applied to the 12 resting-state network probability maps as a positive control, and subsequently to the full PET atlas database as the primary discovery analysis, both in the HCP-YA rsfMRI sample and separately per run. For each analysis, group- and individual-level NEOFC scores and associated p values were derived from the null distributions as described above. To correct for the effective number of simultaneous tests, the M_eff_ was computed from the Pearson intercorrelation matrix of the respective atlas set (resting-state networks or PET) separately for each tested parcellation and applied to group-level p values for both AUC+ and AUC−. Individual-level significance was assessed at uncorrected p < 0.05; the proportion of subjects exceeding this threshold was reported to characterize the consistency of effects across individuals. We reported results for the first rsfMRI run in the results section (all data in supplementary tables).

#### Test-retest analysis

Test-retest reliability of individual-level NEOFC scores was evaluated using the two available rsfMRI runs from the HCP-YA dataset, acquired approximately one day apart. For each reference atlas and each combination of parcellation, NEOFC metric, and testing direction (e.g., AUC+ vs. AUC−), the intraclass correlation coefficient ICC(2,k) (two-way random effects model, average measures, absolute agreement) was calculated using pingouin. As a complementary measure of within-subject variability, the within-subject coefficient of variation (WCV) was computed as the root mean square of individual coefficients of variation across runs. Statistical significance of the WCV was assessed against a permutation null distribution (n = 1,000) generated by randomly exchanging session labels within subjects.

#### Reproducibility analysis and p value meta-analysis

Reproducibility of the group mean AUC profiles across independent samples was assessed in two complementary ways; AUC profiles of HCP-YA and YRSP samples were averaged across runs before analyses. First, pairwise Spearman correlations between the group-mean profiles (robust z-scores) across the PET reference atlases were computed for all dataset pairs. Profile reproducibility was additionally summarized as an intraclass correlation coefficient ICC(3,1), treating reference atlases as targets and datasets as raters, separately for AUC+ and AUC−. Second, to increase statistical power to detect weaker effects, a meta-analysis was performed across the six independent fMRI datasets using Stouffer's method^132^, with sample sizes as weights. The M_eff_ for multiple comparison correction was calculated from the reference atlases as described above and applied to the combined p values.

### fMRI-MEG associations

#### Cross-modal associations across NEOFC profiles

Cross-modal profile associations were assessed in the subset of subjects with both quality-controlled rsfMRI and MEG data available (n = 29; rsfMRI data averaged across runs). We calculated Spearman correlations between rsfMRI and MEG AUC profiles (robust z-scores) across all PET reference atlases on two levels: individually for every of 29 subjects, and on the group-level for the mean AUC (rsfMRI averaged across runs).

#### Cross-modal associations per reference atlas across subjects

Cross-modal associations per reference atlas were assessed by computing, for each PET reference atlas and each MEG frequency band separately, the Spearman correlation between individual rsfMRI (run-averaged) and MEG AUC scores across subjects.

### Brain-regional influence estimation

To identify brain regions and connections most influential to the aggregate NEOFC scores, we performed two complementary leave-one-out analyses. We focused on the main whole-brain parcellation (Schaefer-200 with subcortical parcels) to enable simultaneous inference on both structures. First, in a leave-one-region-out analysis, the AUC was recomputed after excluding all connections involving a given region. Second, in a leave-one-connection-out analysis, individual connections were excluded for a given region. In both cases, the importance score was defined as the difference between the full AUC and the leave-one-out AUC, averaged across subjects. Analyses were conducted separately for AUC+ and AUC−. In supplementary figures, we additionally show the spatial correlation pattern between leave-one-region-out maps and the original atlases after conversion to percentiles.

### Physiological associations

Details on physiological data processing are provided in the Supplementary Methods. Pupil area time series (YRSP, pre-downsampled to 1 Hz) were provided minimally preprocessed and further cleaned using amplitude and velocity filtering^133^. Multiple arousal indices were derived: the pupil unrest index (PUI; root mean squared of first-differences)^134,135^ and power in different frequency bands defined by Rizzuto et al.^136^. Photoplethysmography signals (HCP-YA, 400 Hz) were processed with NeuroKit2 (Elgendi method) to extract RMSSD^137^ and MadNN, averaged across phase-encoding directions within runs. Sessions were excluded if the NeuroKit2 quality index fell below a data-driven threshold or HRV exceeded 150 ms.

Associations between NET AUC+ and physiological measures were tested using linear mixed models (LMMs) with a random intercept per subject to account for repeated observations across runs. Both the NEOFC scores and the physiological variable were z-standardized prior to modeling. For HCP-YA, covariates included mean FD, sex, age, BMI, and global connectivity; for YRSP, mean FD and global connectivity (demographics not available). Models were fitted via maximum likelihood using the Powell optimizer separately for the two physiological outcomes per dataset and per parcellation.

### Pharmacological challenges

Drug effects on NEOFC scores were assessed using LMMs with a random intercept per subject, fitted via maximum likelihood with the Powell optimizer separately for each reference atlas, AUC+ and AUC− scores, and parcellation. Treatment effects were evaluated using Wald Chi^2^ tests. Mean FD and global connectivity were generally included as covariates to account for potential drug-induced differences in motion and general FC strengths. Multiple testing correction was applied across reference atlases per analysis set, with M_eff_ estimated from the Pearson intercorrelation matrix of individual-level AUC scores within each drug sample. All NEOFC scores and continuous covariates were z-standardized prior to modeling.

For MPH (2-level: placebo/MPH) and risperidone (3-level: placebo/low dose/high dose), we evaluated the main effect of treatment. For MPH, only cortical data and no FD information was available. For ketamine and midazolam, each analyzed separately in a 2×2 within-subjects factorial design (treatment: placebo/drug × session: pre/post-infusion), we focused on the treatment × session interaction. A joint 3×2 model (placebo/ketamine/midazolam × session) was additionally fitted to directly compare the two compounds.

### Clinical comparison

Prior to group comparisons, AUC scores were harmonized across the 4 acquisition sites using ComBat as implemented in neuroHarmonize^76,138,139^. Harmonization was performed separately for each parcellation and testing direction, retaining sex, age, mean FD, and global connectivity as biological covariates. The harmonization model was learned on controls and subsequently applied to patients.

Group differences in NEOFC scores between patients and controls were tested using type II analyses of covariance with Tukey HSD post hoc tests separately per reference atlas, testing direction, and parcellation. All NEOFC scores and continuous covariates were z-standardized prior to modeling. Covariates included sex, age, mean FD, global connectivity, and current antipsychotic drug intake in chlorpromazine equivalents. The M_eff_ was estimated from the intercorrelation of individual-level AUC scores across reference atlases within each analysis set. Three sub-analyses were conducted: (i) restricting the patient group to currently unmedicated subjects vs. controls (no drug intake covariate); (ii) comparing controls, currently medicated, and currently unmedicated patients; and (iii) comparing controls, lifetime medicated, and lifetime unmedicated patients.

Association analyses were calculated in the psychosis group only and between NEOFC scores and six clinical and cognitive outcomes: PANSS total, positive, and negative subscales, current antipsychotics dose and duration of exposure, and the NIH cognitive composite score. For non-antipsychotics outcomes, Spearman correlation and partial Spearman correlation were computed in parallel, adjusting for sex, age, mean FD, global connectivity, and current antipsychotics dose. Correlations with antipsychotics outcomes were calculated without the antipsychotics covariate and while excluding subjects with zero exposure. The M_eff_ was estimated accordingly as the product of the effective number of reference atlases and the effective number of outcomes.

## Supplementary Material

Supplement 1

Supplement 2

Supplement 3

Supplement 4

## References

[1] Logothetis N. K. What we can do and what we cannot do with fMRI. Nature 453, 869–878 (2008). 18548064 10.1038/nature06976

[2] Biswal B. B. & Uddin L. Q. The history and future of resting-state functional magnetic resonance imaging. Nature 641, 1121–1131 (2025). 40437164 10.1038/s41586-025-08953-9 PMC12628496

[3] Biswal B., Zerrin Yetkin F., Haughton V. M. & Hyde J. S. Functional connectivity in the motor cortex of resting human brain using echo-planar mri. Magnetic Resonance in Medicine 34, 537–541 (1995). 8524021 10.1002/mrm.1910340409

[4] Damoiseaux J. S. Consistent resting-state networks across healthy subjects. Proceedings of the National Academy of Sciences 103, 13848–13853 (2006). 10.1073/pnas.0601417103 PMC1564249 16945915

[5] Hipp J. F., Hawellek D. J., Corbetta M., Siegel M. & Engel A. K. Large-scale cortical correlation structure of spontaneous oscillatory activity. Nat Neurosci 15, 884–890 (2012). 22561454 10.1038/nn.3101 PMC3861400

[6] Huntenburg J. M., Bazin P.-L. & Margulies D. S. Large-Scale Gradients in Human Cortical Organization. Trends in Cognitive Sciences 22, 21–31 (2018). 29203085 10.1016/j.tics.2017.11.002

[7] Taylor H. P. Functional hierarchy of the human neocortex across the lifespan. Nature 1–10 (2026) doi:10.1038/s41586-026-10219-x. PMC13102691 41882352

[8] Bazinet V., Hansen J. Y. & Misic B. Towards a biologically annotated brain connectome. Nat. Rev. Neurosci. 24, 747–760 (2023). 37848663 10.1038/s41583-023-00752-3

[9] Baillet S. Magnetoencephalography for brain electrophysiology and imaging. Nat Neurosci 20, 327–339 (2017). 28230841 10.1038/nn.4504

[10] Dukart J. Cerebral blood flow predicts differential neurotransmitter activity. Sci Rep 8, 4074 (2018). 29511260 10.1038/s41598-018-22444-0 PMC5840131

[11] Dukart J. JuSpace: A tool for spatial correlation analyses of magnetic resonance imaging data with nuclear imaging derived neurotransmitter maps. Human Brain Mapping 42, 555–566 (2021). 33079453 10.1002/hbm.25244 PMC7814756

[12] Lotter L. D., Nehls S., Losse E., Dukart J. & Chechko N. Temporal dissociation between local and global functional adaptations of the maternal brain to childbirth: A longitudinal assessment. 2023.08.15.553345 Preprint at 10.1101/2023.08.15.553345 (2023). PMC11473773 38769432

[13] Kasper J. Resting state changes in aging and Parkinson’s disease are shaped by underlying neurotransmission – a normative modeling study. 2023.10.18.562677 Preprint at 10.1101/2023.10.18.562677 (2023). 38679325

[14] Kasper J. Local synchronicity in dopamine-rich caudate nucleus influences Huntington’s disease motor phenotype. Brain 146, 3319–3330 (2023). 36795496 10.1093/brain/awad043

[15] Hahn L. Resting-state alterations in behavioral variant frontotemporal dementia are related to the distribution of monoamine and GABA neurotransmitter systems. eLife 13, e86085 (2024). 38224473 10.7554/eLife.86085 PMC10789488

[16] Grumbach P. Local activity alterations in individuals with autism correlate with neurotransmitter properties and ketamine-induced brain changes. Nat Commun 16, 8248 (2025). 40925922 10.1038/s41467-025-63857-6 PMC12420803

[17] Hansen J. Y. Mapping neurotransmitter systems to the structural and functional organization of the human neocortex. Nat Neurosci 25, 1569–1581 (2022). 36303070 10.1038/s41593-022-01186-3 PMC9630096

[18] Hansen J. Y. Integrating multimodal and multiscale connectivity blueprints of the human cerebral cortex in health and disease. PLoS Biol 21, e3002314 (2023). 37747886 10.1371/journal.pbio.3002314 PMC10553842

[19] Saiz-Masvidal C. Mapping cross-modal functional connectivity of major neurotransmitter systems in the human brain. Brain Struct Funct 230, 137 (2025). 40828212 10.1007/s00429-025-02996-4 PMC12364969

[20] Siebenhühner F., Palva J. M. & Palva S. Linking the microarchitecture of neurotransmitter systems to large-scale MEG resting state networks. iScience 27, (2024). 10.1016/j.isci.2024.111111 PMC11544385 39524335

[21] Shafiei G. Neurophysiological signatures of cortical micro-architecture. Nat Commun 14, 6000 (2023). 37752115 10.1038/s41467-023-41689-6 PMC10522715

[22] Dipasquale O. Receptor-Enriched Analysis of functional connectivity by targets (REACT): A novel, multimodal analytical approach informed by PET to study the pharmacodynamic response of the brain under MDMA. NeuroImage 195, 252–260 (2019). 30953835 10.1016/j.neuroimage.2019.04.007 PMC6547164

[23] Cao Z. Unraveling the molecular relevance of brain phenotypes: A comparative analysis of null models and test statistics. NeuroImage 293, 120622 (2024). 38648869 10.1016/j.neuroimage.2024.120622 PMC11132826

[24] Dipasquale O. Unravelling the effects of methylphenidate on the dopaminergic and noradrenergic functional circuits. Neuropsychopharmacol. 45, 1482–1489 (2020). 10.1038/s41386-020-0724-x PMC7360745 32473593

[25] Lotter L. D. & Dukart J. Nispace: Neuroimaging Spatial Colocalization Environment. Zenodo 10.5281/zenodo.12514622 (2024).

[26] Markello R. D. neuromaps: structural and functional interpretation of brain maps. Nat Methods 1–8 (2022) doi:10.1038/s41592-022-01625-w. 36203018 PMC9636018

[27] Markello R. D. & Misic B. Comparing spatial null models for brain maps. NeuroImage 236, 118052 (2021). 33857618 10.1016/j.neuroimage.2021.118052

[28] Dworetsky A. Probabilistic mapping of human functional brain networks identifies regions of high group consensus. NeuroImage 237, 118164 (2021). 34000397 10.1016/j.neuroimage.2021.118164 PMC8296467

[29] Van Essen D. C. The WU-Minn Human Connectome Project: An overview. NeuroImage 80, 62–79 (2013). 23684880 10.1016/j.neuroimage.2013.05.041 PMC3724347

[30] Shafiei G., Baillet S. & Misic B. Human electromagnetic and haemodynamic networks systematically converge in unimodal cortex and diverge in transmodal cortex. PLOS Biology 20, e3001735 (2022). 35914002 10.1371/journal.pbio.3001735 PMC9371256

[31] Lotter L. D. Regional patterns of human cortex development correlate with underlying neurobiology. Nat Commun 15, 7987 (2024). 39284858 10.1038/s41467-024-52366-7 PMC11405413

[32] Schroeter S. Immunolocalization of the cocaine- and antidepressant-sensitive l-norepinephrine transporter. Journal of Comparative Neurology 420, 211–232 (2000). 10753308

[33] Samuels E. R. & Szabadi E. Functional Neuroanatomy of the Noradrenergic Locus Coeruleus: Its Roles in the Regulation of Arousal and Autonomic Function Part I: Principles of Functional Organisation. Current Neuropharmacology 6, 235–253 (2008). 19506723 10.2174/157015908785777229 PMC2687936

[34] Bolt T. Autonomic physiological coupling of the global fMRI signal. Nat Neurosci 28, 1327–1335 (2025). 40335772 10.1038/s41593-025-01945-y PMC13725885

[35] Ciric R. Benchmarking of participant-level confound regression strategies for the control of motion artifact in studies of functional connectivity. NeuroImage 154, 174–187 (2017). 28302591 10.1016/j.neuroimage.2017.03.020 PMC5483393

[36] Lee K. Arousal impacts distributed hubs modulating the integration of brain functional connectivity. NeuroImage 258, 119364 (2022). 35690257 10.1016/j.neuroimage.2022.119364 PMC9341222

[37] Aston-Jones G. & Cohen J. D. An integrative theory of locus coeruleus-norepinephrine function: adaptive gain and optimal performance. Annu Rev Neurosci 28, 403–450 (2005). 16022602 10.1146/annurev.neuro.28.061604.135709

[38] Hawkins P. C. T. The effect of risperidone on reward-related brain activity is robust to drug-induced vascular changes. Human Brain Mapping 42, 2766–2777 (2021). 33666305 10.1002/hbm.25400 PMC8127149

[39] Forsyth A. Comparison of local spectral modulation, and temporal correlation, of simultaneously recorded EEG/fMRI signals during ketamine and midazolam sedation. Psychopharmacology 235, 3479–3493 (2018). 30426183 10.1007/s00213-018-5064-8

[40] Stoof U. M., Friston K. J., Tisdall M., Cooray G. K. & Rosch R. E. Topographic Variation in Human Neurotransmitter Receptor Densities Explains Differences in Intracranial EEG Spectra. Hum Brain Mapp 46, e70393 (2025). 41171139 10.1002/hbm.70393 PMC12576962

[41] Buzsáki G. & Wang X.-J. Mechanisms of Gamma Oscillations. Annu Rev Neurosci 35, 203–225 (2012). 22443509 10.1146/annurev-neuro-062111-150444 PMC4049541

[42] Manning J. R., Jacobs J., Fried I. & Kahana M. J. Broadband Shifts in Local Field Potential Power Spectra Are Correlated with Single-Neuron Spiking in Humans. J. Neurosci. 29, 13613–13620 (2009). 19864573 10.1523/JNEUROSCI.2041-09.2009 PMC3001247

[43] Canolty R. T. & Knight R. T. The functional role of cross-frequency coupling. Trends in Cognitive Sciences 14, 506–515 (2010). 20932795 10.1016/j.tics.2010.09.001 PMC3359652

[44] Engel A. K., Gerloff C., Hilgetag C. C. & Nolte G. Intrinsic Coupling Modes: Multiscale Interactions in Ongoing Brain Activity. Neuron 80, 867–886 (2013). 24267648 10.1016/j.neuron.2013.09.038

[45] Uddin L. Q. Salience processing and insular cortical function and dysfunction. Nat Rev Neurosci 16, 55–61 (2015). 25406711 10.1038/nrn3857

[46] Shine J. M. Neuromodulatory Influences on Integration and Segregation in the Brain. Trends in Cognitive Sciences 23, 572–583 (2019). 31076192 10.1016/j.tics.2019.04.002

[47] Kristensen A. S. SLC6 neurotransmitter transporters: structure, function, and regulation. Pharmacol Rev 63, 585–640 (2011). 21752877 10.1124/pr.108.000869

[48] Siegel M., Donner T. H. & Engel A. K. Spectral fingerprints of large-scale neuronal interactions. Nat Rev Neurosci 13, 121–134 (2012). 22233726 10.1038/nrn3137

[49] Logothetis N. K., Pauls J., Augath M., Trinath T. & Oeltermann A. Neurophysiological investigation of the basis of the fMRI signal. Nature 412, 150–157 (2001). 11449264 10.1038/35084005

[50] Hipp J. F. & Siegel M. BOLD fMRI Correlation Reflects Frequency-Specific Neuronal Correlation. Current Biology 25, 1368–1374 (2015). 25936551 10.1016/j.cub.2015.03.049

[51] Brookes M. J. Investigating the electrophysiological basis of resting state networks using magnetoencephalography. Proceedings of the National Academy of Sciences 108, 16783–16788 (2011). 10.1073/pnas.1112685108 PMC3189080 21930901

[52] Sastre-Yagüe D. Cortical excitability inversely modulates fMRI connectivity via low-frequency neuronal coupling. 2026.03.12.710517 Preprint at 10.64898/2026.03.12.710517 (2026).

[53] Schotte A. Risperidone compared with new and reference antipsychotic drugs: in vitro and in vivo receptor binding. Psychopharmacology 124, 57–73 (1996). 8935801 10.1007/BF02245606

[54] Nyberg S., Farde L., Eriksson L., Halldin C. & Eriksson B. 5-HT2 and D2 dopamine receptor occupancy in the living human brain. Psychopharmacology 110, 265–272 (1993). 7530376 10.1007/BF02251280

[55] Mion G. & Villevieille T. Ketamine Pharmacology: An Update (Pharmacodynamics and Molecular Aspects, Recent Findings). CNS Neuroscience & Therapeutics 19, 370–380 (2013). 23575437 10.1111/cns.12099 PMC6493357

[56] Wang J., Sun P. & Liang P. Neuropsychopharmacological effects of midazolam on the human brain. Brain Inf. 7, 15 (2020). 10.1186/s40708-020-00116-y PMC7655878 33170396

[57] Keiser M., Hasan M. & Oswald S. Affinity of Ketamine to Clinically Relevant Transporters. Mol. Pharmaceutics 15, 326–331 (2018). 10.1021/acs.molpharmaceut.7b00627 29191019

[58] Kegeles L. S. Modulation of amphetamine-induced striatal dopamine release by ketamine in humans: implications for schizophrenia. Biological Psychiatry 48, 627–640 (2000). 11032974 10.1016/s0006-3223(00)00976-8

[59] Meyer T. Predictive value of heart rate in treatment of major depression with ketamine in two controlled trials. Clin Neurophysiol 132, 1339–1346 (2021). 33888426 10.1016/j.clinph.2021.01.030

[60] Zhu J., Spencer T. J., Liu-Chen L.-Y., Biederman J. & Bhide P. G. Methylphenidate and μ opioid receptor interactions: A pharmacological target for prevention of stimulant abuse. Neuropharmacology 61, 283–292 (2011). 21545805 10.1016/j.neuropharm.2011.04.015 PMC3105120

[61] Batistela S., Bueno O. F. A., Vaz L. J. & Galduróz J. C. F. Methylphenidate as a cognitive enhancer in healthy young people. Dement Neuropsychol 10, 134–142 (2016). 29213444 10.1590/S1980-5764-2016DN1002009 PMC5642404

[62] Pourhamzeh M. The Roles of Serotonin in Neuropsychiatric Disorders. Cell Mol Neurobiol 42, 1671–1692 (2021). 33651238 10.1007/s10571-021-01064-9 PMC11421740

[63] Tost H., Alam T. & Meyer-Lindenberg A. Dopamine and Psychosis: Theory, Pathomechanisms and Intermediate Phenotypes. Neurosci Biobehav Rev 34, 689–700 (2010). 19559045 10.1016/j.neubiorev.2009.06.005 PMC2838993

[64] Royer J. Opportunities and pitfalls of data contextualization in neuroimaging. Nat. Rev. Neurosci. 1–19 (2026) doi:10.1038/s41583-026-01038-0. 41927918

[65] Hansen J. Y. & Misic B. Integrating and interpreting brain maps. Trends in Neurosciences 48, 594–607 (2025). 40744776 10.1016/j.tins.2025.06.003

[66] Hansen J. Y. Inter-individual variability of neurotransmitter receptor and transporter density in the human brain. Brain Struct Funct 231, 13 (2026). 41528514 10.1007/s00429-025-03069-2 PMC12799681

[67] Glasser M. F. The minimal preprocessing pipelines for the Human Connectome Project. Neuroimage 80, 105–124 (2013). 23668970 10.1016/j.neuroimage.2013.04.127 PMC3720813

[68] Fischl B. FreeSurfer. Neuroimage 62, 774–781 (2012). 22248573 10.1016/j.neuroimage.2012.01.021 PMC3685476

[69] Esteban O. fMRIPrep: a robust preprocessing pipeline for functional MRI. Nat Methods 16, 111–116 (2019). 30532080 10.1038/s41592-018-0235-4 PMC6319393

[70] Mehta K. XCP-D: A robust pipeline for the post-processing of fMRI data. Imaging Neuroscience 2, imag–2–00257 (2024). 10.1162/imag_a_00257 PMC12288603 40800264

[71] Harris C. R. Array programming with NumPy. Nature 585, 357–362 (2020). 32939066 10.1038/s41586-020-2649-2 PMC7759461

[72] McKinney W. Data Structures for Statistical Computing in Python. in 56–61 (Austin, Texas, 2010). doi:10.25080/Majora-92bf1922-00a.

[73] Virtanen P. SciPy 1.0: fundamental algorithms for scientific computing in Python. Nat Methods 17, 261–272 (2020). 32015543 10.1038/s41592-019-0686-2 PMC7056644

[74] Seabold S. & Perktold J. Statsmodels: Econometric and Statistical Modeling with Python. SciPy 2010 https://doi.org/10.25080/Majora-92bf1922-011 (2010) doi:10.25080/Majora-92bf1922-011.

[75] Vallat R. Pingouin: statistics in Python. Journal of Open Source Software 3, 1026 (2018).

[76] Pomponio R. Harmonization of large MRI datasets for the analysis of brain imaging patterns throughout the lifespan. NeuroImage 208, 116450 (2020). 31821869 10.1016/j.neuroimage.2019.116450 PMC6980790

[77] Abraham A. Machine learning for neuroimaging with scikit-learn. Frontiers in Neuroinformatics 8, (2014). 10.3389/fninf.2014.00014 PMC3930868 24600388

[78] Makowski D. NeuroKit2: A Python toolbox for neurophysiological signal processing. Behav Res 53, 1689–1696 (2021). 10.3758/s13428-020-01516-y 33528817

[79] Hunter J. D. Matplotlib: A 2D Graphics Environment. Computing in Science Engineering 9, 90–95 (2007).

[80] Waskom M. L. seaborn: statistical data visualization. Journal of Open Source Software 6, 3021 (2021).

[81] Castrillon G. An energy costly architecture of neuromodulators for human brain evolution and cognition. Science Advances 9, eadi7632 (2023). 38091393 10.1126/sciadv.adi7632 PMC10848727

[82] Finnema S. J. Imaging synaptic density in the living human brain. Sci Transl Med 8, 348ra96 (2016). 10.1126/scitranslmed.aaf6667 27440727

[83] Finnema S. J. Kinetic evaluation and test-retest reproducibility of [11C]UCB-J, a novel radioligand for positron emission tomography imaging of synaptic vesicle glycoprotein 2A in humans. J Cereb Blood Flow Metab 38, 2041–2052 (2018). 28792356 10.1177/0271678X17724947 PMC6259313

[84] Andersson J. D., Matuskey D. & Finnema S. J. Positron emission tomography imaging of the γ-aminobutyric acid system. Neuroscience Letters 691, 35–43 (2019). 30102960 10.1016/j.neulet.2018.08.010

[85] Chen M.-K. Comparison of [11C]UCB-J and [18F]FDG PET in Alzheimer’s disease: A tracer kinetic modeling study. J Cereb Blood Flow Metab 41, 2395–2409 (2021). 33757318 10.1177/0271678X211004312 PMC8393289

[86] Chen M.-K. Assessing Synaptic Density in Alzheimer Disease With Synaptic Vesicle Glycoprotein 2A Positron Emission Tomographic Imaging. JAMA Neurol 75, 1215 (2018). 30014145 10.1001/jamaneurol.2018.1836 PMC6233853

[87] Holmes S. E. Lower synaptic density is associated with depression severity and network alterations. Nat Commun 10, 1529 (2019). 30948709 10.1038/s41467-019-09562-7 PMC6449365

[88] Wey H.-Y. Insights into neuroepigenetics through human histone deacetylase PET imaging. Sci. Transl. Med. 8, (2016). 10.1126/scitranslmed.aaf7551 PMC5784409 27510902

[89] Larsen B. Maturation of the human striatal dopamine system revealed by PET and quantitative MRI. Nat Commun 11, 846 (2020). 32051403 10.1038/s41467-020-14693-3 PMC7015913

[90] Luna B. PETfrog. Openneuro 10.18112/OPENNEURO.DS002385.V1.1.0 (2021).

[91] Smart K. Sex differences in [11C]ABP688 binding: a positron emission tomography study of mGlu5 receptors. Eur J Nucl Med Mol Imaging 46, 1179–1183 (2019). 30627817 10.1007/s00259-018-4252-4 PMC6451701

[92] Galovic M. Validation of a combined image derived input function and venous sampling approach for the quantification of [18F]GE-179 PET binding in the brain. NeuroImage 237, 118194 (2021). 34023451 10.1016/j.neuroimage.2021.118194

[93] Galovic M. In Vivo *N*-Methyl- d -Aspartate Receptor (NMDAR) Density as Assessed Using Positron Emission Tomography During Recovery From NMDAR-Antibody Encephalitis. JAMA Neurol 80, 211 (2023). 36469313 10.1001/jamaneurol.2022.4352 PMC9857588

[94] Nørgaard M. A high-resolution in vivo atlas of the human brain’s benzodiazepine binding site of GABAA receptors. Neuroimage 232, 117878 (2021). 33610745 10.1016/j.neuroimage.2021.117878 PMC8256681

[95] Lukow P. B. Cellular and molecular signatures of in vivo imaging measures of GABAergic neurotransmission in the human brain. Commun Biol 5, 1–11 (2022). 35440709 10.1038/s42003-022-03268-1 PMC9018713

[96] Hesse S. Central noradrenaline transporter availability in highly obese, non-depressed individuals. Eur J Nucl Med Mol Imaging 44, 1056–1064 (2017). 28066877 10.1007/s00259-016-3590-3 PMC5538358

[97] Hillmer A. T. Imaging of cerebral α4β2* nicotinic acetylcholine receptors with (-)-[(18)F]Flubatine PET: Implementation of bolus plus constant infusion and sensitivity to acetylcholine in human brain. Neuroimage 141, 71–80 (2016). 27426839 10.1016/j.neuroimage.2016.07.026 PMC5026941

[98] Baldassarri S. R. Use of Electronic Cigarettes Leads to Significant Beta2-Nicotinic Acetylcholine Receptor Occupancy: Evidence From a PET Imaging Study. Nicotine & Tobacco Research 20, 425–433 (2018). 28460123 10.1093/ntr/ntx091 PMC5896427

[99] Naganawa M. First in Human Assessment of the Novel M1 Muscarinic Acetylcholine Receptor PET Radiotracer 11C-LSN3172176. Journal of Nuclear Medicine https://doi.org/10.2967/jnumed.120.246967 (2020) doi:10.2967/jnumed.120.246967. PMC8049371 32859711

[100] Aghourian M. Quantification of brain cholinergic denervation in Alzheimer’s disease using PET imaging with [18F]-FEOBV. Mol Psychiatry 22, 1531–1538 (2017). 28894304 10.1038/mp.2017.183

[101] García Gómez F., Huertas I., Lojo Ramírez J. & García Solís D. Elaboración de una plantilla de SPM para la normalización de imágenes de PET con 18F-DOPA. ImagenDiagnostica 9, 23–25 (2018).

[102] Kaller S. Test-retest measurements of dopamine D1-type receptors using simultaneous PET/MRI imaging. Eur J Nucl Med Mol Imaging 44, 1025–1032 (2017). 28197685 10.1007/s00259-017-3645-0

[103] Sandiego C. M. Reference region modeling approaches for amphetamine challenge studies with [11C]FLB 457 and PET. J Cereb Blood Flow Metab 35, 623–629 (2015). 25564239 10.1038/jcbfm.2014.237 PMC4420880

[104] Sandiego C. M. Imaging robust microglial activation after lipopolysaccharide administration in humans with PET. Proceedings of the National Academy of Sciences 112, 12468–12473 (2015). 10.1073/pnas.1511003112 PMC4603509 26385967

[105] Sandiego C. M. The Effect of Treatment with Guanfacine, an Alpha2 Adrenergic Agonist, on Dopaminergic Tone in Tobacco Smokers: An [11C]FLB457 PET Study. Neuropsychopharmacol. 43, 1052–1058 (2018). 10.1038/npp.2017.223 PMC5854798 28944773

[106] Beliveau V. A High-Resolution In Vivo Atlas of the Human Brain’s Serotonin System. J Neurosci 37, 120–128 (2017). 28053035 10.1523/JNEUROSCI.2830-16.2016 PMC5214625

[107] Radhakrishnan R. Age-Related Change in 5-HT6 Receptor Availability in Healthy Male Volunteers Measured with 11C-GSK215083 PET. J Nucl Med 59, 1445–1450 (2018). 29626125 10.2967/jnumed.117.206516 PMC6126437

[108] Radhakrishnan R. In vivo 5-HT6 and 5-HT2A receptor availability in antipsychotic treated schizophrenia patients vs. unmedicated healthy humans measured with [11C]GSK215083 PET. Psychiatry Research: Neuroimaging 295, 111007 (2020). 31760336 10.1016/j.pscychresns.2019.111007

[109] Kantonen T. Interindividual variability and lateralization of μ-opioid receptors in the human brain. NeuroImage 217, 116922 (2020). 32407992 10.1016/j.neuroimage.2020.116922

[110] Vijay A. PET imaging reveals lower kappa opioid receptor availability in alcoholics but no effect of age. Neuropsychopharmacol 43, 2539–2547 (2018). 10.1038/s41386-018-0199-1 PMC6224533 30188515

[111] Normandin M. D. Imaging the cannabinoid CB_1_ receptor in humans with [11C]OMAR: assessment of kinetic analysis methods, test-retest reproducibility, and gender differences. J Cereb Blood Flow Metab 35, 1313–1322 (2015). 25833345 10.1038/jcbfm.2015.46 PMC4528005

[112] D’Souza D. C. Rapid Changes in Cannabinoid 1 Receptor Availability in Cannabis-Dependent Male Subjects After Abstinence From Cannabis. Biological Psychiatry: Cognitive Neuroscience and Neuroimaging 1, 60–67 (2016). 10.1016/j.bpsc.2015.09.008 PMC4742341 29560896

[113] Ranganathan M. Reduced Brain Cannabinoid Receptor Availability in Schizophrenia. Biological Psychiatry 79, 997–1005 (2016). 26432420 10.1016/j.biopsych.2015.08.021 PMC4884543

[114] Neumeister A. Positron Emission Tomography Shows Elevated Cannabinoid CB1 Receptor Binding in Men with Alcohol Dependence. Alcoholism Clin & Exp Res 36, 2104–2109 (2012). 10.1111/j.1530-0277.2012.01815.x PMC3418442 22551199

[115] Hoffmann M., Hoopes A., Greve D. N., Fischl B. & Dalca A. V. Anatomy-aware and acquisition-agnostic joint registration with SynthMorph. Imaging Neuroscience 2, imag–2–00197 (2024). 10.1162/imag_a_00197 PMC11247402 39015335

[116] Sydnor V. J. Intrinsic Activity Develops Along a Sensorimotor-Association Cortical Axis in Youth. 2022.08.15.503994 Preprint at 10.1101/2022.08.15.503994 (2022).

[117] Paquola C. The BigBrainWarp toolbox for integration of BigBrain 3D histology with multimodal neuroimaging. eLife 10, e70119 (2021). 34431476 10.7554/eLife.70119 PMC8445620

[118] Amunts K. BigBrain: An Ultrahigh-Resolution 3D Human Brain Model. Science 340, 1472–1475 (2013). 23788795 10.1126/science.1235381

[119] Lorio S. New tissue priors for improved automated classification of subcortical brain structures on MRI. NeuroImage 130, 157–166 (2016). 26854557 10.1016/j.neuroimage.2016.01.062 PMC4819722

[120] Huck J. High resolution atlas of the venous brain vasculature from 7 T quantitative susceptibility maps. Brain Struct Funct 224, 2467–2485 (2019). 31278570 10.1007/s00429-019-01919-4

[121] Mouches P. & Forkert N. D. A statistical atlas of cerebral arteries generated using multi-center MRA datasets from healthy subjects. Sci Data 6, 29 (2019). 30975990 10.1038/s41597-019-0034-5 PMC6472360

[122] Schaefer A. Local-Global Parcellation of the Human Cerebral Cortex from Intrinsic Functional Connectivity MRI. Cereb Cortex 28, 3095–3114 (2018). 28981612 10.1093/cercor/bhx179 PMC6095216

[123] Lee Kangjoo Yale Resting State fMRI/Pupillometry: Arousal Study. Openneuro 10.18112/OPENNEURO.DS003673.V2.0.1 (2022).

[124] Jacobs G. R. An Introduction to the Human Connectome Project for Early Psychosis. Schizophrenia Bulletin sbae 123 (2024) doi:10.1093/schbul/sbae123. PMC12061660 39036958

[125] Larabi D. I., Gell M., Amico E., Eickhoff S. B. & Patil K. R. Spatially localized fMRI metrics as predictive and highly distinct state-independent fingerprints. 2021.08.03.454862 Preprint at 10.1101/2021.08.03.454862 (2022).

[126] Tadel F., Baillet S., Mosher J. C., Pantazis D. & Leahy R. M. Brainstorm: A User-Friendly Application for MEG/EEG Analysis. Computational Intelligence and Neuroscience 2011, 879716 (2011). 21584256 10.1155/2011/879716 PMC3090754

[127] Bruns A., Eckhorn R., Jokeit H. & Ebner A. Amplitude envelope correlation detects coupling among incoherent brain signals. Neuroreport 11, 1509–1514 (2000). 10841367

[128] Alexander-Bloch A. F. On testing for spatial correspondence between maps of human brain structure and function. Neuroimage 178, 540–551 (2018). 29860082 10.1016/j.neuroimage.2018.05.070 PMC6095687

[129] Vos de Wael R. BrainSpace: a toolbox for the analysis of macroscale gradients in neuroimaging and connectomics datasets. Commun Biol 3, 1–10 (2020). 32139786 10.1038/s42003-020-0794-7 PMC7058611

[130] Wagner H. H. & Dray S. Generating spatially constrained null models for irregularly spaced data using Moran spectral randomization methods. Methods in Ecology and Evolution 6, 1169–1178 (2015).

[131] Li J. & Ji L. Adjusting multiple testing in multilocus analyses using the eigenvalues of a correlation matrix. Heredity 95, 221–227 (2005). 16077740 10.1038/sj.hdy.6800717

[132] Whitlock M. C. Combining probability from independent tests: the weighted Z-method is superior to Fisher’s approach. Journal of Evolutionary Biology 18, 1368–1373 (2005). 16135132 10.1111/j.1420-9101.2005.00917.x

[133] Kret M. E. & Sjak-Shie E. E. Preprocessing pupil size data: Guidelines and code. Behav Res 51, 1336–1342 (2019). 10.3758/s13428-018-1075-y PMC6538573 29992408

[134] Lüdtke H., Wilhelm B., Adler M., Schaeffel F. & Wilhelm H. Mathematical procedures in data recording and processing of pupillary fatigue waves. Vision Research 38, 2889–2896 (1998). 9797985 10.1016/s0042-6989(98)00081-9

[135] Wilhelm B., Wilhelm H., Lüdtke H., Streicher P. & Adler M. Pupillographic assessment of sleepiness in sleep-deprived healthy subjects. Sleep 21, 258–265 (1998). 9595604

[136] Rizzuto V. Pupillary Hippus as a Biomarker: Spectral Signatures and Complexity Approaches in Autonomic and Clinical Contexts. Bioengineering (Basel) 12, 1376 (2025). 41463672 10.3390/bioengineering12121376 PMC12729395

[137] Heart rate variability: standards of measurement, physiological interpretation and clinical use. Task Force of the European Society of Cardiology and the North American Society of Pacing and Electrophysiology. Circulation 93, 1043–1065 (1996). 8598068

[138] Johnson W. E., Li C. & Rabinovic A. Adjusting batch effects in microarray expression data using empirical Bayes methods. Biostatistics 8, 118–127 (2007). 16632515 10.1093/biostatistics/kxj037

[139] Fortin J.-P. Harmonization of cortical thickness measurements across scanners and sites. NeuroImage 167, 104–120 (2018). 29155184 10.1016/j.neuroimage.2017.11.024 PMC5845848

