# Supplementary figures and images for: Linking human brain functional connectivity to underlying neurotransmission
Source: bioRxiv. 2026 May 5:2026.04.28.721294. Preprint. [Version 2] doi: 10.64898/2026.04.28.721294 (PMC13142320; doi:10.64898/2026.04.28.721294)

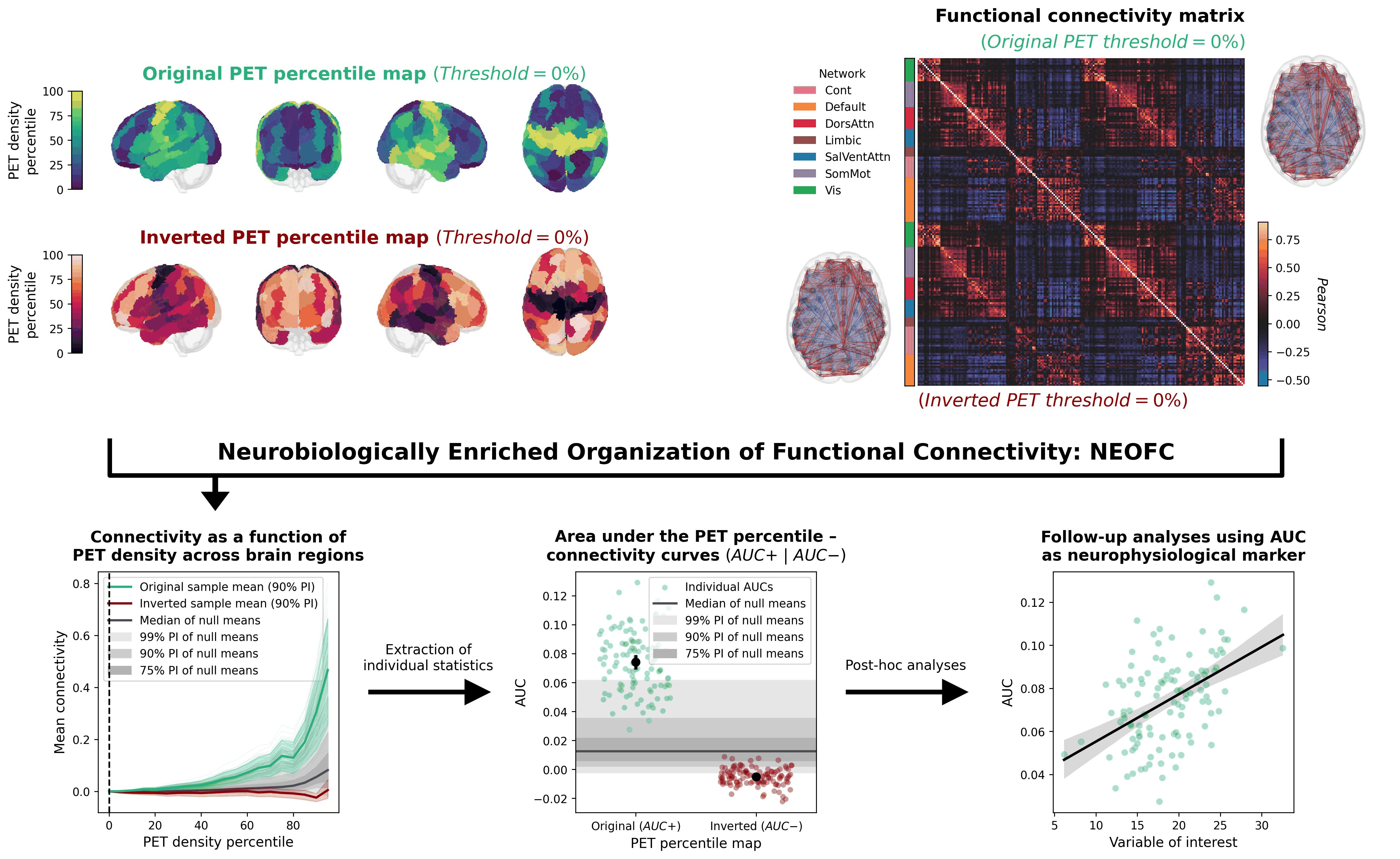

Supplement: Supplement 4 [file media-4.gif]
